# Combination of Lactate and Base Deficit Levels at Admission to Predict Mortality in Blunt Trauma Patients

**DOI:** 10.7759/cureus.40097

**Published:** 2023-06-07

**Authors:** Candace L Ward, Samantha N Olafson, Ryan B Cohen, Mark J Kaplan, Alexi Bloom, Afshin Parsikia, Benjamin J Moran, Pak S Leung

**Affiliations:** 1 Surgery, Einstein Medical Center Philadelphia, Philadelphia, USA; 2 General Surgery, Einstein Medical Center Philadelphia, Philadelphia, USA; 3 Trauma and Acute Care Surgery, Einstein Healthcare Network, Philadelphia, USA; 4 Trauma and Acute Care Surgery, Einstein Medical Center Philadelphia, Philadelphia, USA; 5 General Surgery, Einstein Healthcare Network, Philadelphia, USA

**Keywords:** predictive model, base deficit, blunt force trauma, trauma resuscitation, blood lactate levels

## Abstract

Introduction: Elevated lactate levels are associated with increased mortality in both trauma and non-trauma patients. The relation between base deficit (BD) and mortality is less clear. Traumatologists debate the utility of elevated lactate (EL) versus BD in predicting mortality. We hypothesized that EL (2mmol/L to 5mmol/L) and BD (≤−2mmol/L) in combination could predict mortality in blunt trauma patients.

Methods: This is a retrospective analysis of the trauma registry from 2012 to 2021 at a level 1 trauma center. Blunt trauma patients with admission lactate and BD values were included in the analysis. Exclusion criteria were age <18, penetrating trauma, unknown mortality, and unknown lactate or BD. Logistics regression of the total 5153 charts showed 93% of the patients presented with lactate levels <5mmol/L, therefore patients with lactate >5mmol/L were excluded as outliers. The primary outcome was mortality.

Results: A total of 4794 patients (151 non-survivors) were included in the analysis. Non-survivors had higher rates of EL + BD (35.8% vs. 14.4%, p <0.001). When comparing survivors and non-survivors, EL + BD (OR 5.69), age >65 (5.17), injury severity score (ISS) >25 (8.87), Glasgow coma scale <8 (8.51), systolic blood pressure (SBP) <90 (4.2), and ICU admission (2.61) were significant predictors of mortality. Other than GCS <8 and ISS >25, EL + BD had the highest odds of predicting mortality.

Conclusion: Elevated lactate + BD on admission in combination represents a 5.6-fold increase in mortality in blunt trauma patients and can be used to predict a patient's outcome on admission. This combination variable provides an additional early data point to identify patients at elevated risk of mortality at the moment of admission.

## Introduction

Physiologic disruption can be measured with serum acid and base levels; elevated lactate levels (EL) are associated with increased mortality in both trauma and non-trauma patients [1-3]. Physiologically, EL is a tissue byproduct from anaerobic metabolism due to poor perfusion and decreased tissue oxygenation. Shock states reduce tissue perfusion and therefore tissue oxygenation, forcing cells to resort to anaerobic metabolism to generate energy, creating lactic acid as a byproduct. Lactate clearance theory posits that as lactate levels decrease, tissue perfusion concomitantly improves. Lactate clearance has been investigated as a predictor for survival in critically ill patients [2]. Elevated lactate is directly correlated with injury severity as well as increased mortality risk in trauma patients [4]. One single-center study from Germany revealed a two- to seven-fold increased risk of mortality in patients with lactate above 2mmol/L [1]. In the non-trauma population, this holds true, with 30-day mortality rates of 28.4% to 69% in patients with lactate greater than 4mmol/L. Similarly, Chebl et. al demonstrated that lactate greater than 4mmol/L on presentation had a seven- to 29-fold increased risk of in-hospital mortality over levels below 4mmol/L [5]. In patients with isolated thoracic trauma, Çinar et. al found a direct correlation between EL and mortality risk, reporting a 1.19 times higher risk of mortality per millimole of lactate above normal [2].

The relation between base deficit (BD) and mortality is less clear. Traumatologists debate the utility of EL versus BD in predicting mortality [3,5-7]. One large study of 2271 trauma patients concluded that BD was a better predictor of mortality with an odds ratio of 1.1 [6]. In contrast to lactate, a BD may be more readily attainable, as they are calculated values correlating well with measured bicarbonate levels [7]. Many studies have evaluated the utility of BD as a factor in trauma evaluations, although these findings vary in correlation with mortality rate [7-11]. There are currently no definitive guidelines on the utilization of BD alone for blunt trauma patients. One multicenter study compared admission lactate and BD levels in relation to mortality in blunt trauma patients. Both EL and BD were initially independently associated with mortality, however, when early deaths were excluded (within 24 hours), the relationship was lost [10]. A German study noted that BD better correlated with in-hospital mortality, need for blood product transfusion, and massive transfusion rates. Similar to lactate clearance, change in BD levels over time may be a better predictor of resuscitative success and development of multi-organ failure and mortality [6,12].

Investigation into EL and BD levels thus far has been in isolation, i.e., analyzing each factor’s impact on mortality individually. As both factors paint a picture of the physiologic upset within a patient, we sought to similarly evaluate the bigger picture of the non-penetrating trauma patient. While these laboratory values are associated with patients at increased risk of mortality, we hypothesized that EL (2mmol/L to 5mmol/L) and BD (≤−2mmol/l) in combination could better predict mortality in blunt trauma patients.

## Materials and methods

**Figure 1:**
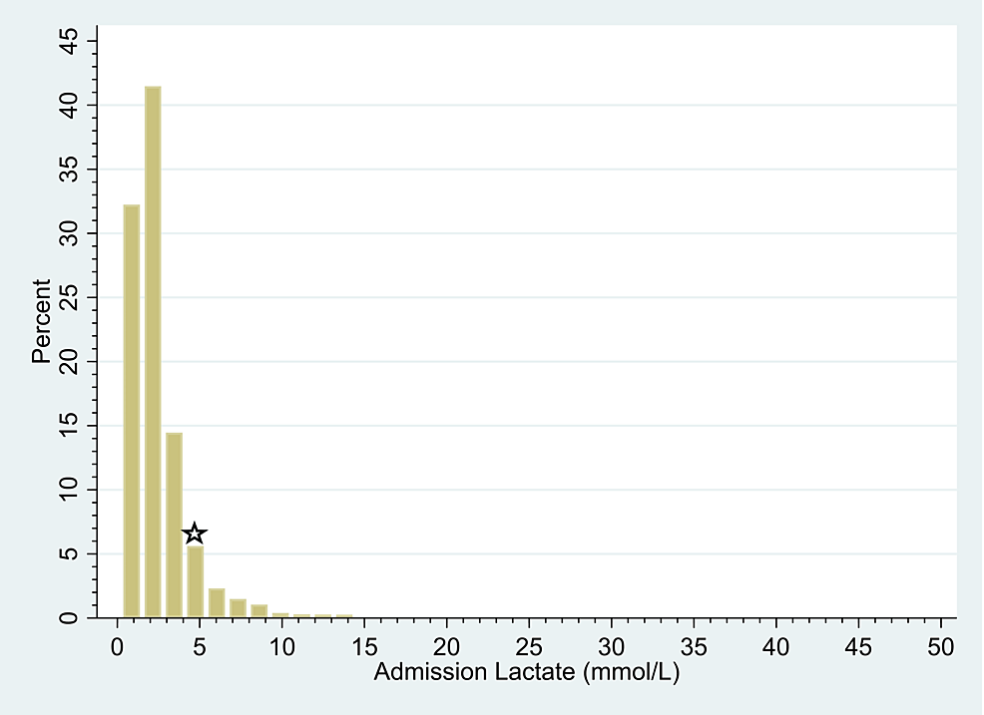
Distribution of lactate levels on admission The cutoff value is marked by the star

This is a 10-year retrospective review from 2012 to 2021 at an urban level 1 trauma center. After obtaining approval from the Institutional Review Board of Einstein Healthcare Network, Philadelphia, USA (approval no. IRB-2022-883), the trauma registry was queried for blunt trauma patients with both admission lactate and BD values. Per trauma protocol, these values are drawn upon presentation to the trauma bay, usually within 15 minutes of presentation. Patients who were under 18, had a penetrating mechanism, unknown mortality, or no lactate or BD levels were excluded. Patient demographics were collected, as well as injury severity score (ISS), presenting Glasgow coma scale (GCS), and ICU admission. A total of 5183 charts meeting these criteria were found. Initial analysis showed that the majority (93%) of patients had lactate levels less than 5mmol/L, thus patients with lactate levels above 5mmol/L were outliers and were excluded (see Figure 1).

The primary outcome was mortality during admission. The differences between alive and dead patients were evaluated. Subsequently, a logistic multivariate regression helped in evaluating the combined role of EL and BD to predict mortality. 

## Results

A total of 4794 patients met the inclusion criteria. Of which, 151 non-survivors were identified. Non-survivors were overall older, more likely to be above the age of 65, and more likely to be white (Table 1). There was a similar distribution of gender between the two groups.

**Table 1:** Trauma patients who survived vs. succumbed EL: Elevated lactate (>2mmol/L), BD: Base deficit, BE: Base excess, ISS: Injury severity score, GCS: Glasgow coma scale, SBP: Systolic blood pressure, HR: Heart rate, AIS: Abbreviated injury scale * Indicates AIS ≥3; Max acceptable lactate was set as ≤5mmol/L

Variables	Mortality (no), n=4643	Mortality (yes), n=151	p-value
Age	57.23 (21.3)	71.1 (17.7)	<0.001
Age >65	1748 (37.6%)	98 (64.9%)	<0.001
Admission lactate, median (IQR)*	1.887 (1.3 to 2.6)	2.442 (1.6 to 3.33)	<0.001
Admission BD, median (IQR)	0.3 (−1.7 to 2.4)	−0.9 (−4.1 to 2.2)	<0.001
Gender (male)	2670 (57.5%)	95 (62.9%)	0.19
Race (white)	1369 (31.6%)	62 (44.6%)	0.001
ISS, median (IQR)	9 (5 to 13)	17 (10 to 26)	<0.001
ISS 1-15 (mild)	3852 (83.0%)	65 (43.0%)	<0.001
ISS 15-25 (moderate)	585 (12.6%)	42 (27.8%)	<0.001
ISS >25 (severe)	206 (4.4%)	44 (29.1%)	<0.001
GCS, median (IQR)	15 (15 to 15)	14 (6 to 15)	<0.001
GCS ≤8	145 (3.1%)	52 (34.4%)	<0.001
GCS 9-12	157 (3.4%)	14 (9.3%)	<0.001
GCS 13-15	4341 (93.5%)	85 (56.3%)	<0.001
SBP <90	49 (1.1%)	15 (9.9%)	<0.001
HR >100	1020 (22.0%)	40 (26.5%)	0.19
ICU admission	2526 (54.4%)	138 (91.4%)	<0.001
Not EL and normal acid-base balance	1345 (29.0%)	14 (9.3%)	<0.001
BD only	320 (6.9%)	13 (8.6%)	0.41
BE only	928 (20.0%)	26 (17.2%)	0.40
EL only	1021 (22.0%)	30 (19.9%)	0.53
EL+BD	669 (14.4%)	54 (35.8%)	<0.001
EL+BE	360 (7.8%)	14 (9.3%)	0.49
Pre-hospital intubation	25 (0.5%)	17 (11.4%)	<0.001
Severe head injury*	1316 (28.3%)	103 (68.2%)	<0.001
Severe chest injury*	734 (15.8%)	33 (21.9%)	0.046
Severe abdomen injury*	141 (3.0%)	5 (3.3%)	0.85

When comparing survivors and non-survivors, non-survivors had worse injuries and physiology. Survivors’ median admission lactate was 1.8 in comparison to 2.4 for non-survivors (p <0.001). Survivors’ median BD was 0.3 in contrast to −0.9 for non-survivors (p <0.001). The ISS for non-survivors was higher with a higher rate of head injuries and pre-hospital intubation, and they were more likely to be admitted to ICU, have a lower GCS, and have hypotension on presentation.

When comparing survivors and non-survivors, EL + BD (OR 5.69, p <0.001), age greater than 65 (OR 5.17, p <0.001), ISS greater than 25 (OR 8.87, p <0.001), GCS less than 8 (OR 8.51, p <0.001), SBP <90 (OR 4.2, p <0.001), and ICU admission (OR 2.61, p <0.001) were significant predictors of mortality (see Table 2).

**Table 2:** Odds ratios for variable predictability of mortality. Values in bold denote statistically significant predictors. EL: Elevated lactate (>2mmol/L), BD: Base deficit, BE: Base excess, ISS: Injury severity score, GCS: Glasgow coma scale, SBP: Systolic blood pressure, HR: Heart rate, AIS: Abbreviated injury scale * Indicates AIS ≥ 3; Max acceptable lactate was set as ≤5mmol/L

Variables	Odds ratio	SE	p-value	95% CI lower	95% CI upper
Age >65	5.173	1.234	<0.001	3.241	8.257
Race (white)	1.787	0.361	0.004	1.203	2.654
ISS category					
1-8	Reference				
9-15	1.461	0.518	0.285	0.729	2.928
16-25	3.006	1.241	0.008	1.339	6.750
>25	8.872	4.107		3.581	21.981
GCS category					
GCS 13-15	Reference				
GCS ≤8	8.515	2.560	<0.001	4.723	15.350
GCS 9-12	2.091	0.735	0.036	1.050	4.164
SBP <90	4.219	1.725	<0.001	1.893	9.403
ICU admission	2.615	0.857	0.003	1.375	4.971
Lactate and acid-base balance (individually or in combination)					
EL + BD	5.693	1.983	<0.001	2.877	11.266
Pre-hospital intubation	0.997	0.501	0.995	0.372	2.670
Severe head injury*	1.218	0.359	0.504	0.683	2.171
Severe chest injury*	0.862	0.245	0.601	0.494	1.503

**Figure 2:**
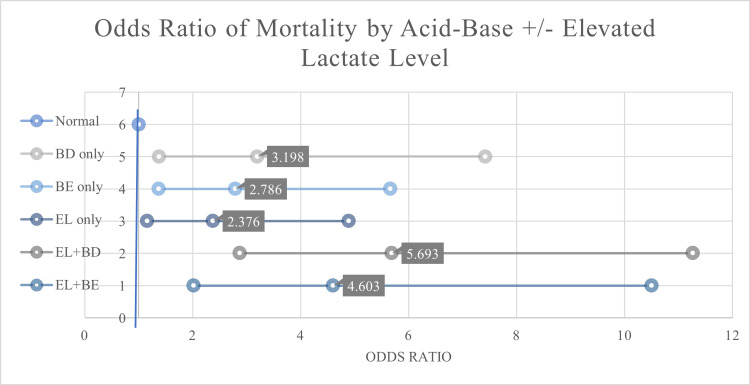
Odds ratios based on acid-base status and lactate levels All variables were found to be significant with p <0.05 EL: Elevated lactate (>2mmol/L), BD: Base deficit, BE: Base excess

When evaluating BD, base excess, and EL levels, two values were found to be significant. Patients who had normal acid-base balance and normal lactate had a higher rate of survival, as anticipated. Individual variables of BD, base excess, EL level, or EL with base excess were all found to be statistically similar between our two groups. However, the combination variable of BD with EL was found to be statistically significant between survivors and non-survivors. Non-survivors had higher rates of EL + BD (35.8% vs. 14.4%, p <0.001). Other than GCS below 8 and ISS above 25, EL + BD had the highest odds of predicting mortality with an odds ratio of 5.6 (see Figure 2).

## Discussion

This study analyzed the utility of combining admission lactate with BD to predict outcomes in blunt trauma patients. A 5.6-fold increased odds of mortality was found with both EL and BD in the blunt trauma population.

Elevated lactate remains a well-established independent predictor of mortality, correlating with increased risks by a factor of 2 to 29 [1,13,14]. During times of stress and poor tissue perfusion, anaerobic metabolism begins, producing lactate acid. As shock is a marker of poor tissue production, EL level is a widely accepted surrogate marker of shock and is used to guide resuscitation [8,15,16]. A recent publication in the trauma literature describes the utility of age-adapted lactate values to improve the predictive value in older patients [17].

In contrast to the measured value of lactic acid, BD is a calculated value based on bicarbonate, pH, and the amount of acid needed to titrate one liter of blood to a normal pH [16]. Due to this tabulated value, it may be more readily available compared to serum lactate. The BD does have shortcomings; as a formulated value, it is an indirect estimate of acidosis due to poor tissue perfusion. There is debate over whether external factors such as alcohol levels and over-resuscitation with crystalloids may impair results [18,19]. As such, ongoing debate exists about the validity and reliability of BD as an individual predictor of mortality in trauma patients, and thus there is no concrete guidance on its utilization [16,20]. Several large studies have found that an excessive BD (<−6) was predictive of worse outcomes in blunt trauma patients.

Numerous statistical models exist to summarize the risk of mortality in trauma patients. Several rely on diagnoses and studies performed during the patient's hospital stay and are therefore not quickly available. The trauma and injury severity score (TRISS), developed in the early 1980s, utilizes the revised trauma score, ISS, and the patient's age with a specificity of 97.5% and sensitivity of 21.95% [21]. After being used for decades, recent studies have found that TRISS overestimates trauma mortality; therefore, additional methods of prediction need to be investigated. The trauma early mortality prediction tool (TEMPT) was developed at the University of Southern California to identify trauma patients at increased risk for mortality without relying on the abbreviated injury scale (AIS) [22]. The variables comprising this tool include the presence of traumatic brain injury (TBI), age (≥59.5 years), BD (≤−4.35 mmol/L), prothrombin time (PTT; ≥31.45 seconds), international normalized ratio (INR; ≥1.25), and temperature (≤36.25°C). Identifying at-risk patients provides the opportunity to stratify risk and target research into improving trauma outcomes.

This is the first study to evaluate both EL and BD to predict mortality in blunt trauma patients. Our study evaluated these two markers of physiologic upset in conjunction as a novel way of viewing the entire chemical physiologic picture of a trauma patient. Our findings recognize that the combination of both EL and BD is predictive of mortality and can identify patients at high risk upon admission. Blunt trauma patients may be under-triaged due to the absence of obvious external trauma; therefore, a variable that more rapidly identifies patients at risk of mortality can alert the provider to more serious underlying physiologic disruption. We suggest that, if the patient has both a significant BD and EL, the provider pays closer attention to the patient's resuscitative needs and more closely monitors this patient, possibly admitting them to a higher level of care. Predictive models have shifted away from using AIS as a variable for mortality prediction due to its retrospective nature. The combination variable EL + BD offers similar value by providing risk stratification at presentation rather than at the conclusion of the patient’s course. These patients may require more aggressive fluid resuscitation, and we plan to investigate lactate clearance rates as well as blood transfusion rates in these patients. 

The strength of this study lies in its single-center data source. This study analyzed nearly 5000 patients over a decade using laboratory values conducted at the same lab, decreasing the risk of measurement bias. Limitations include the sampling of similar patients at a single trauma center and the exclusion of lactate levels above 5 mmol/L. Blunt trauma patients alone were evaluated due to the increased management difficulty and frequency of under-triaging blunt injuries. Single-center studies increase the risk of sampling bias. However, this sample is large and generalizable to many major urban centers. By excluding patients with lactate levels above 5, we attempted to eliminate outliers, which, however, may have dampened our calculated odds ratio or significance by doing so.

## Conclusions

Injury severity score is a strong predictor of mortality but is not available at the time of admission. The combination of EL + BD on admission represents a 5.6-fold increase in mortality in blunt trauma patients and can be used to predict a patient’s outcome on admission. We can use this value to quickly identify trauma patients at increased risk upon arrival and assess their needs to improve outcomes. Patients with EL and significant BD upon arrival may warrant closer scrutiny and more aggressive resuscitation. Further research into the utility of this combination of variables as a predictor for other trauma outcomes is warranted.

## References

[1] Bernhard M, Döll S, Kramer A, Weidhase L, Hartwig T, Petros S, Gries A (2020). Elevated admission lactate levels in the emergency department are associated with increased 30-day mortality in non-trauma critically ill patients. Scand J Trauma Resusc Emerg Med.

[2] Çınar E, Usul E, Demirtaş E, Gökçe A (2021). The role of trauma scoring systems and serum lactate level in predicting prognosis in thoracic trauma. Ulus Travma Acil Cerrahi Derg.

[3] Huh Y, Ko Y, Hwang K (2021). Admission lactate and base deficit in predicting outcomes of pediatric trauma. Shock.

[4] Hagebusch P, Faul P, Klug A, Gramlich Y, Hoffmann R, Schweigkofler U (2022). Elevated serum lactate levels and age are associated with an increased risk for severe injury in trauma team activation due to trauma mechanism. Eur J Trauma Emerg Surg.

[5] Bou Chebl R, El Khuri C, Shami A, Rajha E, Faris N, Bachir R, Abou Dagher G (2017). Serum lactate is an independent predictor of hospital mortality in critically ill patients in the emergency department: a retrospective study. Scand J Trauma Resusc Emerg Med.

[6] Davis JW, Sue LP, Dirks RC (2020). Admission base deficit is superior to lactate in identifying shock and resuscitative needs in trauma patients. Am J Surg.

[7] Eachempati SR, Robb T, Ivatury RR, Hydo LJ, Barie PS (2002). Factors associated with mortality in patients with penetrating abdominal vascular trauma. J Surg Res.

[8] Gale SC, Kocik JF, Creath R, Crystal JS, Dombrovskiy VY (2016). A comparison of initial lactate and initial base deficit as predictors of mortality after severe blunt trauma. J Surg Res.

[9] Rutherford EJ, Morris JA Jr, Reed GW, Hall KS (1992). Base deficit stratifies mortality and determines therapy. J Trauma.

[10] Mutschler M, Nienaber U, Brockamp T (2013). Renaissance of base deficit for the initial assessment of trauma patients: a base deficit-based classification for hypovolemic shock developed on data from 16,305 patients derived from the TraumaRegister DGU®. Crit Care.

[11] Siegel JH, Rivkind AI, Dalal S, Goodarzi S (1990). Early physiologic predictors of injury severity and death in blunt multiple trauma. Arch Surg.

[12] Kincaid EH, Miller PR, Meredith JW, Rahman N, Chang MC (1998). Elevated arterial base deficit in trauma patients: a marker of impaired oxygen utilization. J Am Coll Surg.

[13] Kramer A, Urban N, Döll S (2019). Early lactate dynamics in critically ill non-traumatic patients in a resuscitation room of a German emergency department (OBSERvE-Lactate-Study). J Emerg Med.

[14] Callaway DW, Shapiro NI, Donnino MW, Baker C, Rosen CL (2009). Serum lactate and base deficit as predictors of mortality in normotensive elderly blunt trauma patients. J Trauma.

[15] Richards JE, Mazzeffi MA, Massey MS, Rock P, Galvagno SM, Scalea TM (2021). The bitter and the sweet: relationship of lactate, glucose, and mortality after severe blunt trauma. Anesth Analg.

[16] Qi J, Bao L, Yang P, Chen D (2021). Comparison of base excess, lactate and pH predicting 72-h mortality of multiple trauma. BMC Emerg Med.

[17] Hagebusch P, Faul P, Ruckes C (2022). The predictive value of serum lactate to forecast injury severity in trauma-patients increases taking age into account. Eur J Trauma Emerg Surg.

[18] Gustafson ML, Hollosi S, Chumbe JT, Samanta D, Modak A, Bethea A (2015). The effect of ethanol on lactate and base deficit as predictors of morbidity and mortality in trauma. Am J Emerg Med.

[19] Dunne JR, Tracy JK, Scalea TM, Napolitano LM (2005). Lactate and base deficit in trauma: does alcohol or drug use impair their predictive accuracy?. J Trauma.

[20] Ibrahim I, Chor WP, Chue KM, Tan CS, Tan HL, Siddiqui FJ, Hartman M (2016). Is arterial base deficit still a useful prognostic marker in trauma? A systematic review. Am J Emerg Med.

[21] Singh J, Gupta G, Garg R, Gupta A (2011). Evaluation of trauma and prediction of outcome using TRISS method. J Emerg Trauma Shock.

[22] Kunitake RC, Kornblith LZ, Cohen MJ, Callcut RA (2018). Trauma early mortality prediction tool (TEMPT) for assessing 28-day mortality. Trauma Surg Acute Care Open.

